# Pulsatilla in Sleeplessness

**Published:** 1895-09

**Authors:** 


### PULSATILLA IN SLEEPLESSNESS.

A hard-worked bookkeeper of fifty-six years complained of restless sleep with anxious dreams of his work, which he is unable to accomplish. Indoors he feels oppressed, even when the air is not warm ; constant occipital headache, worse at night in bed. Though he feels no pain anywhere, he is generally depressed and weeps easily ; but this does not relieve. He is rather inclined to obesity and of a blonde type. Pulsatilla$^{200}$, two drops in fourteen days. After the second dose he felt greatly relieved and felt entirely well without taking any more treatment.—*A. N. Z.*, 21, 91.

